# Scleredema Diabeticorum: A Rare Metabolic Connective Tissue Manifestation of Type 2 Diabetes Mellitus Causing External Restrictive Lung Disease

**DOI:** 10.7759/cureus.60374

**Published:** 2024-05-15

**Authors:** Madhushan Ranabahu, Wasantha Karunarathna, Jayanjana Asanthi, Dinesh Dassanayake, Hemal Senanayake

**Affiliations:** 1 Medicine, Postgraduate Institute of Medicine, University of Colombo, Anuradhapura, LKA; 2 Pathology, Teaching Hospital Anuradhapura, Anuradhapura, LKA; 3 Pulmonary Medicine, Teaching Hospital Anuradhapura, Anuradhapura, LKA; 4 Medicine, Faculty of Medicine, University of Rajarata, Anuradhapura, LKA

**Keywords:** advanced glycation end products, collagen fiber, spirometry, obesity, diabetes mellitus

## Abstract

Scleredema diabeticorum (SD) is a rare metabolic connective tissue manifestation of diabetes mellitus (DM). SD commonly manifests in male patients with poorly controlled prolonged DM with obesity. In SD, the skin gets stiffened, thickened, and leathery in texture with a peau d’orange appearance commonly involving the posterior aspect of the neck and chest wall. Extensive chest wall skin involvement restricts lung movement, causing external restrictive lung disease and hypoventilation. In this case report, we present a 50-year-old male patient with poorly controlled type 2 DM for 10 years, complicated with established diabetic microvascular complications and extensive involvement of SD over the back of the neck and chest with external restrictive lung disease.

## Introduction

Diabetes mellitus (DM) is a common multisystem metabolic disorder. The prevalence of DM in adults is 23.0% in Sri Lanka [1]. Poorly controlled diabetes can lead to arterial vascular endothelial dysfunction, causing large-to-medium arterial atheroma formation and small capillary arteriolar vasculopathy. Scleredema of Buschke is a rare connective tissue disease used to describe distinct histopathological changes occurring in the dermis and subcutaneous tissue [2]. There are three types described in the literature according to etiology. Type 1 scleredema (55%) is associated with streptococcal infection [2], type 2 scleredema (25%) is associated with paraproteinemia secondary to hematological malignancy [2], and type 3 is (3%) is scleredema diabeticorum (SD) [2]. SD is a rare complication of diabetes, which is mainly seen in type 2 DM but occasionally in type 1 DM as well [3]. Long-established poorly controlled DM with microvascular complications, obesity, and male gender are the recognized risk factors for SD [3]. SD mainly affects the skin. However, internal organ involvement has also been reported. Skin lesions are commonly seen in the upper body, especially on the back of the neck, scalp, upper chest, and shoulders, and are rarely seen on the thighs and face [4]. SD seldom involves internal organs such as the heart, lungs, muscles, intestines, salivary glands, and synovial joints (frozen shoulder) [4]. Recognized manifestations of SD include external restrictive lung disease, diabetic cheiroarthropathy, disfiguration of the face, ophthalmoplegia, slurring of speech, and restriction in joint mobility [4-6]. There are no identified treatments for SD. However, good diabetic control and weight reduction might reduce the progression of the disease and sometimes cure it [2,4]. Immune-modulatory drugs such as methotrexate, cyclosporine, and topical steroids may be beneficial [2].

## Case presentation

**Figure 1:**
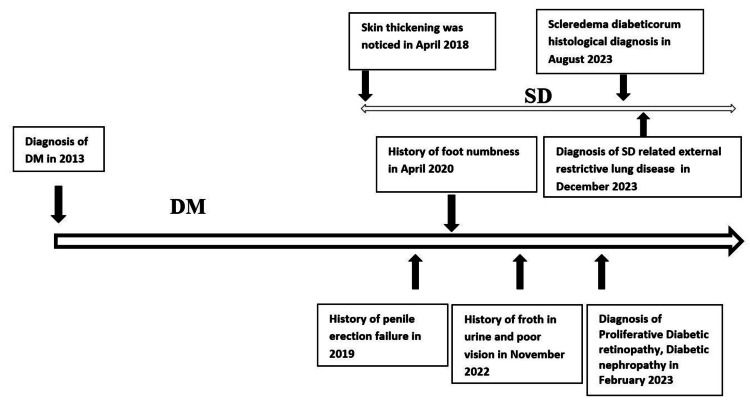
A graphical representation of the case showing how type 2 diabetes mellitus (DM) progressed to microvascular complications parallel with scleredema diabeticorum (SD).

A 49-year-old male patient who was diagnosed with poorly controlled type 2 DM in 2013 complained of thickening and stiffening of the skin over the posterior aspect of the neck, scalp, and upper chest since 2018. He noticed that the skin changed its stiffness and distribution gradually over time. However, skin lesions did not cause symptoms. He had difficulty in penile erection since 2019 without an early morning erection. The patient had developed numbness and loss of sensation over the bilateral soles of his feet since April 2020. The patient had been complaining of poor vision since November 2022, along with foamy urine, which was later attributed to proliferative diabetic retinopathy and diabetic nephropathy with stage 3b and A2 chronic kidney disease, respectively (Figure 1).

**Figure 2:**
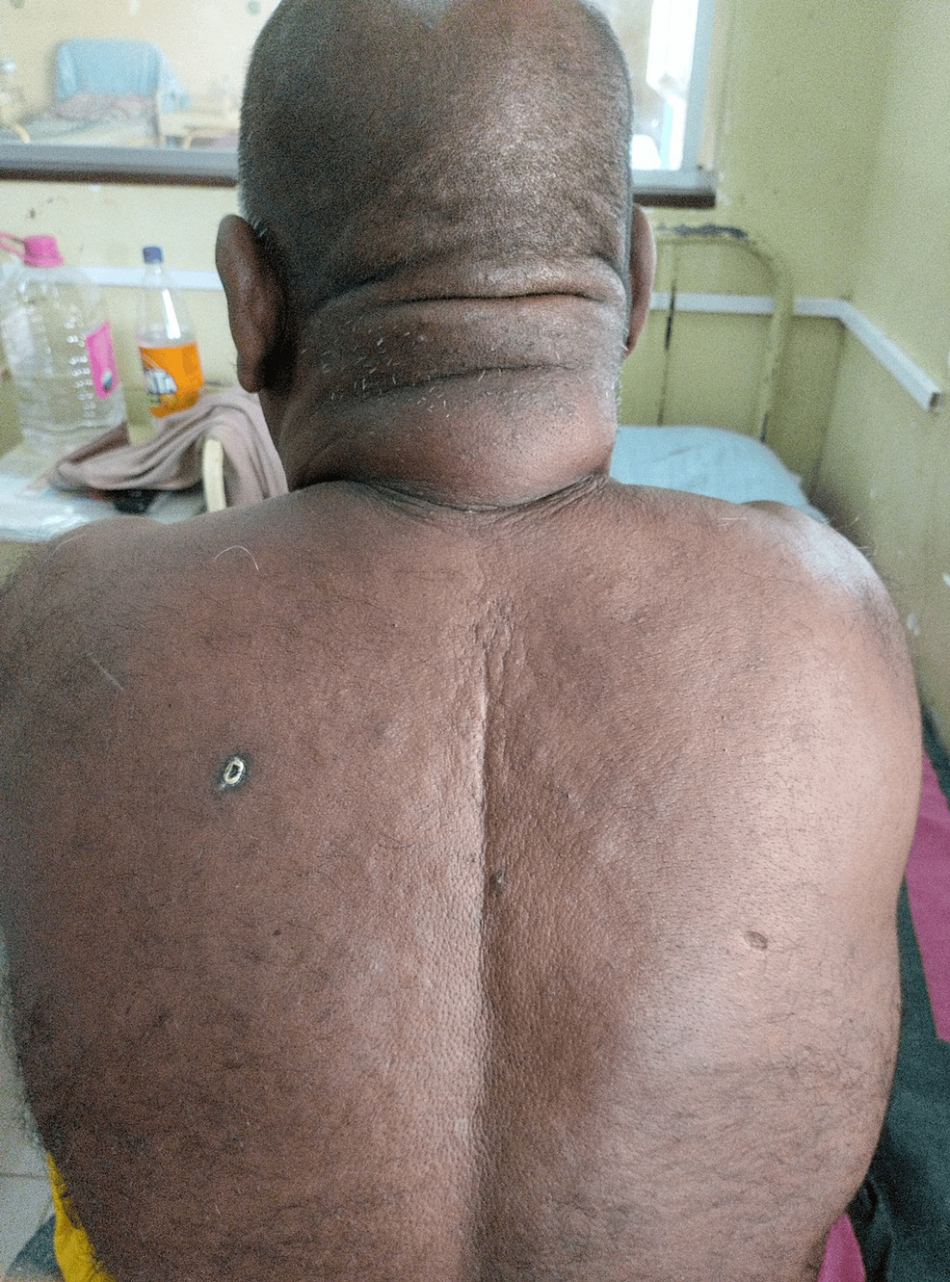
Image of the back of the neck and upper chest showing skin thickening, a peau d’orange appearance, and hair loss on the affected skin area with a shiny appearance.

He had been taking metformin and gliclazide after the diagnosis of DM. However, he did not have a proper medical follow-up. Physical examination revealed a body mass index of 36.3 kg/m^2^, acanthosis nigricans over the neck, and multiple skin tags over bilateral axillae. The skin over the neck, chest, and scalp over the occipital area was extensively indurated with a peau d’orange appearance. The skin texture was thick, leathery, and edematous (Figure 2).

**Figure 3:**
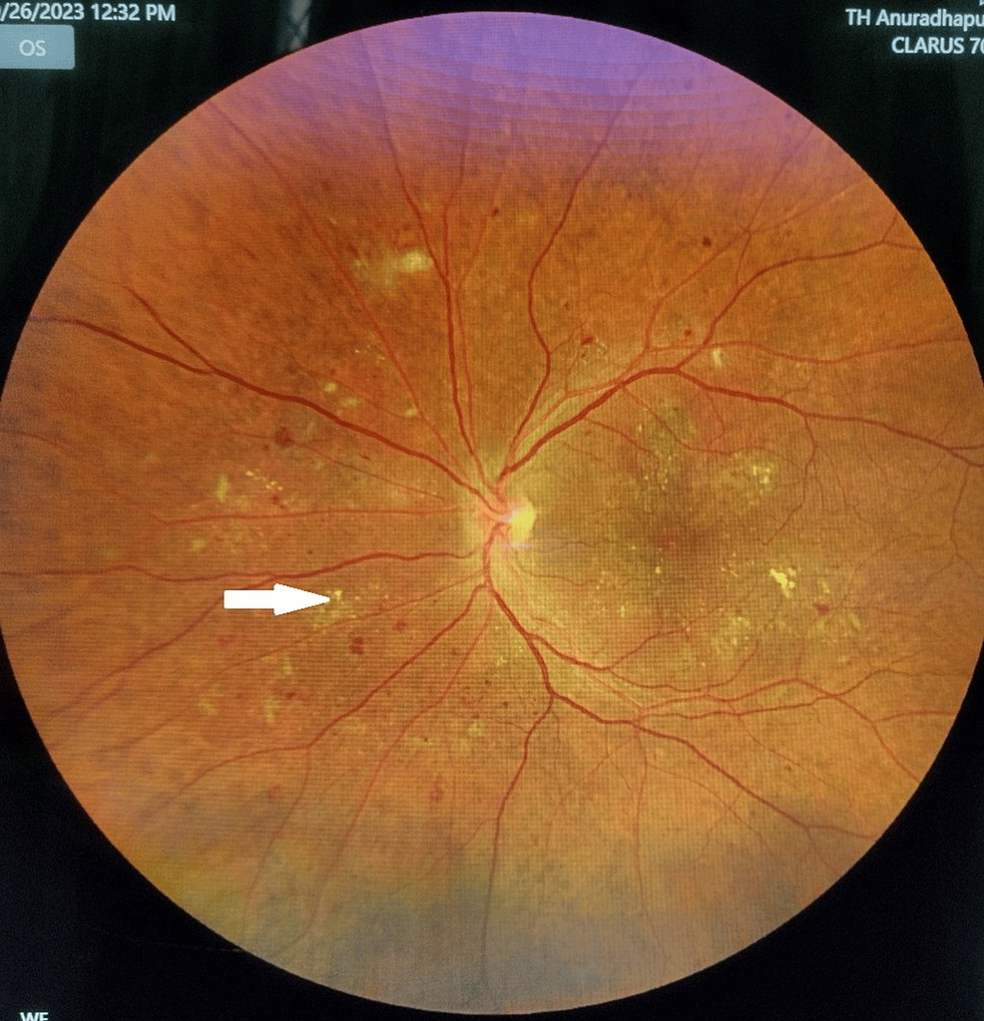
The patient’s left retina showing proliferative diabetic retinopathy. The white arrow shows focal laser treatment areas.

His blood pressure was 140/ 90 mmHg, respiratory rate was 18 breaths per minute in ambient air, trachea was midline, and auscultation revealed vesicular breathing throughout the lung fields. Chest wall expansion was measured at the nipple level. It was 113.5 cm in deep expiration and 116 cm in deep inspiration. The patient’s six-minute walking test showed no pulse oximeter desaturation in ambient air, and SpO_2_ remained at 98%. Fundoscopy examination revealed proliferative diabetic retinopathy with focal laser treatment areas (Figure 3).

**Figure 4:**
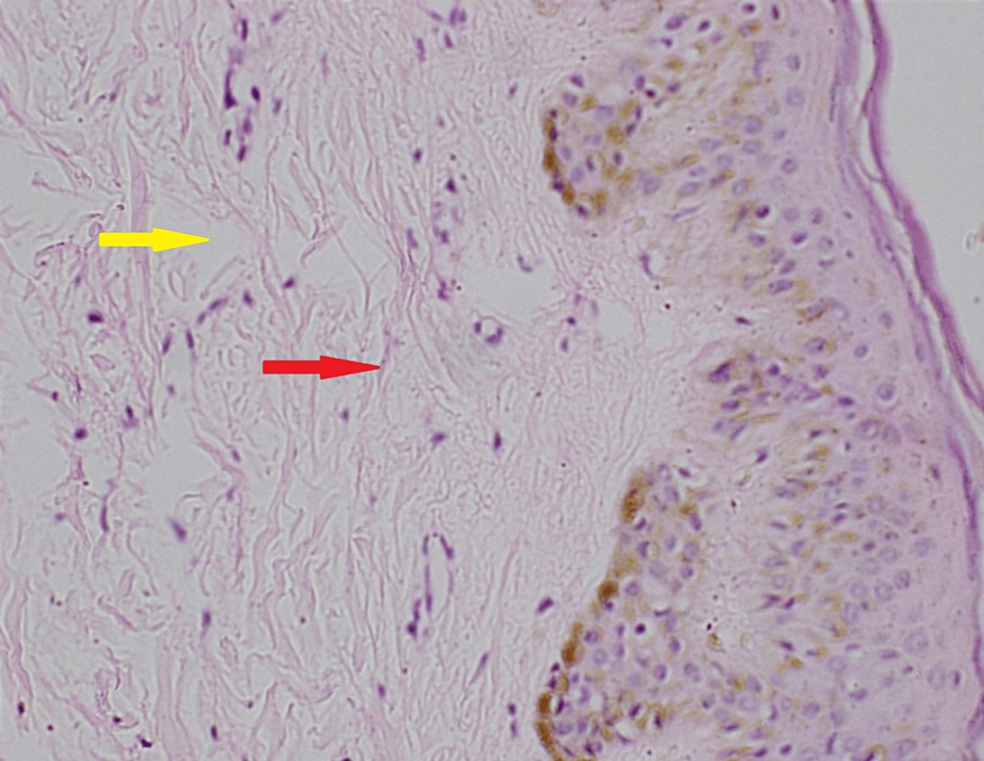
Skin histology. The hematoxylin and eosin stain of the skin biopsy shows increased dermis volume due to increased collagen fibers and mucinous material. The red arrow shows type 1 collagen fibers, and the yellow arrow shows mucinous materials.

The skin biopsy from the posterior aspect of the left chest wall showed chronic perivascular lymphocytic cell infiltration with increased collagen and mucinous material within the dermis. Epidermis and skin appendages were histologically normal (Figure 4). The histology confirmed the diagnosis of scleredema. The patient was evaluated for causes of scleredema. After extensive evaluation, the diagnosis was concluded as SD (Table 1). The two-dimensional echocardiogram showed normal biventricular systolic and diastolic functions and valves without evidence of pulmonary arterial hypertension. High-resolution computed tomography showed normal lung parenchyma. Table 2 depicts the lung function studies. The patient was prescribed oral medications including metformin 500 mg twice daily, gliclazide 40 mg twice daily, empagliflozin 10 mg mane, sitagliptin 50 mg mane, atorvastatin 20 mg nocte, and losartan 50 mg twice daily. Repeat glycated hemoglobin (HbA1C) was 6.8% in four months. However, SD did not clinically improve in the six-month follow-up despite better diabetic control.

**Table 1:** Investigations. HbA1C = glycated hemoglobin; UACR= urine albumin creatinine ratio; pCO_2_ = partial pressure of carbon dioxide; pO_2_ = partial pressure of oxygen; HCO_3-_ = serum bicarbonate; LVH = left ventricular hypertrophy

Investigations	Results	Reference range
White blood cells count	9.8	4–10 × 10^9^/L
Hemoglobin	11.4	11.5–15 g/dL
Platelets	356	150–450 × 10^9^/L
HbA1c	8.4%	<6.5%
Erythrocyte sedimentation rate	50	<20 mm/hour
Urine Bence Jones protein	Negative	-
Serum protein electrophoresis	No monoclonal band	-
Thyroid-stimulating hormone	2.16	0.3–4.2 µIU/mL
Anti-nuclear antibody	Negative	-
Anti-Scl, 70 antibody	Negative	-
Anti-centromere antibody	Negative	-
Anti-Ds DNA antibody	Negative	-
Rheumatoid factor	0.52	<8 IU/mL
UACR	74.86	<30 mg/g
Serum creatinine	186	60–115 µmol/L
Aspartate transaminase	12	10–35 U/L
Alanine transaminase	28	10–40 U/L
Serum albumin	42	35–52 g/L
Serum globulin	35	25–35 g/L
Bone marrow biopsy	No abnormal plasma cell proliferation	-
Electrocardiography	Sinus rhythm, no LVH, or P pulmonale	-
Non-contrast CT neck	Normal cervical vertebrae appearance and alignment	-
Blood pH	7.41	7.35–7.45
pCO_2_ (mmHg)	27.6	35–45 mmHg
pO_2 _	99.9	75–100 mmHg
HCO_3_^- ^	18.9	22–26 mmol/L

**Table 2:** Lung function test. DLCO = carbon monoxide defusing capacity; FEV1 = forced expiratory volume in one second; FVC = forced vital capacity; Kco = transfer coefficient for carbon monoxide; sb = single breath; TLC = total lung capacity; VA = alveolar volume

Parameters	Lower limit of normal	Predicted value	Test value	Predicted (%)
FVC (L)	2.82	3.56	2.10	59.0
FEV_1 _(L)	2.25	2.90	1.84	63.0
FVC/FEV_1_	0.71	0.81	0.87	108.0
TLC sb (L)	4.55	5.7	3.44	51.0
VA sb (L)	4.40	5.55	2.86	50.0
DLCO (mL/minute/mmHg)	23.0	25.2	9.9	39.2
Kco (mL[CO]/minute/L)	3.87	4.54	3.69	81.2

## Discussion

SD is a rare skin manifestation of diabetes, which can be recognized in 2.5%-14% of patients with poorly controlled disease [2]. Poorly controlled DM causes the production of advanced glycation end products, which damage the microvascular endothelium, causing endothelial dysfunction and leaky capillaries [2]. Irreversible glycation of type 1 collagen, microvascular exudation, and microvascular arteriolar thrombosis are the pathologies that occur in the connective tissue [2]. Histology of SD shows a normal epidermis with a thickened dermis, which contains thickened type 1 collagen fibers with reduced reticulin fiber content [2]. Mucopolysaccharide content is increased in the dermis between collagen fibers [2]. There is no proliferation of fibroblast in the dermis in SD [2]. These histological changes are distinct from those of scleroderma, which is an autoimmune connective disorder. Skin histology in scleroderma shows excessive fibroblast proliferation and extensive deposition of new type 1 collagen fibers [5].

This patient had poorly controlled type 2 DM, complicated by established microvascular complications such as stocking-type peripheral sensory polyneuropathy, proliferative diabetic retinopathy, and diabetic nephropathy without clinical manifestations of macrovascular complications. SD skin lesions emerged insidiously over the years. SD clinically presented in this patient as discomfort in neck extension and extensive chest wall involvement (Figure 2).

Chest wall expansion depends on the strength of the inspiratory external intercostal muscle action against lung parenchymal and chest wall resistance. A healthy individual’s maximum chest wall expansion depends on gender, age, body mass index, and height and is 4-7 cm [7]. This patient’s maximum chest wall expansion at the nipple level was 2.5 cm. The pulmonary function test revealed a restrictive lung disease pattern (Table 2). Forced vital capacity (FVC) of 59% predicted and forced expiratory volume in one second/FVC of 108% predicated [8], total lung capacity was 3.44 liters (51% predicted), suggestive of reduced lung volumes without obstructive pathology [8]. Carbon monoxide defusing capacity (DLCO) depends on two factors, namely, the alveolar ventilation and the transfer coefficient for carbon monoxide [9]. DLCO was 39.2% of what was predicted in this patient. The patient’s Kco was normal (81.2%), which suggested normal gas exchange at the alveolar-capillary membrane [9]. The reduction of DLCO was mainly due to a significant decrease in alveolar ventilation. Alveolar volume in this patient was 50% predicated [9]. The reasons for the reduction in alveolar ventilation in this patient included SD, chest wall restriction, and obesity. Therefore, the patient had an external pattern of restrictive lung disease rather than a parenchymal disease (intrinsic) due to SD and obesity.

Three case reports in the literature have discussed external restrictive lung disease due to SD [10-12]. Two patients were male, and the other was female. Common characteristics were age over 50 years with poorly controlled type 2 DM (HbA1C >8.5%), established microvascular complications, and obesity. Two patients had systemic hypertension [10,11]. Two patients had obstructive sleep apnea [10,12].

## Conclusions

SD is a metabolic connective tissue disorder caused by microvascular disease in poorly controlled DM. SD skin manifestations are rare. Disability occurs according to the site, extent, and organ involvement, causing functional impairment. Extensive involvement of the skin over the chest can lead to extrinsic restrictive pattern lung disease and hypoventilation.

## References

[1] Rannan-Eliya RP, Wijemunige N, Perera P (2023). Prevalence of diabetes and pre-diabetes in Sri Lanka: a new global hotspot-estimates from the Sri Lanka Health and Ageing Survey 2018/2019. BMJ Open Diabetes Res Care.

[2] Pereira M, Pinheiro RR, Lencastre A, Bártolo E (2022). Scleredema diabeticorum. Dermatol Reports.

[3] Kyriakou A, Zagalioti SC, Lazaridou E, Patsatsi A (2021). Scleredema diabeticorum - a case report. J Family Med Prim Care.

[4] Sreela L, Sharma R, Sudheesh M (2015). An unusual presentation of a rare disorder-scleredema of Buschke. J Adv Med Dent Sci Res.

[5] Bobeica C, Niculet E, Craescu M (2023). Immunologic and nonimmunologic sclerodermal skin conditions - review. Front Immunol.

[6] Miyares FJ, Kuriakose R, Deleu DT, El-Wahad NA, Al-Hail H (2008). Scleredema diabeticorum with unusual presentation and fatal outcome. Indian J Dermatol.

[7] Debouche S, Pitance L, Robert A, Liistro G, Reychler G (2016). Reliability and reproducibility of chest wall expansion measurement in young healthy adults. J Manipulative Physiol Ther.

[8] Paraskeva MA, Borg BM, Naughton MT (2011). Spirometry. Aust Fam Physician.

[9] Nguyen LP, Harper RW, Louie S (2016). Using and interpreting carbon monoxide diffusing capacity (Dlco) correctly. Consultant.

[10] Alsaeedi SH, Lee P (2010). Treatment of scleredema diabeticorum with tamoxifen. J Rheumatol.

[11] García-Arpal M, Bujalance-Cabrera C, Banegas-Illescas ME, Sánchez-Caminero MP, González-Ruiz L, Villasanti-Rivas N (2021). Scleredema diabeticorum in a patient: un uncommon etiology of restrictive lung pattern. Dermatol Online J.

[12] Bowen AR, Smith L, Zone JJ (2003). Scleredema adultorum of Buschke treated with radiation. Arch Dermatol.

